# Diagnostic Radiation Exposure of Injury Patients in the Emergency Department: A Cross-Sectional Large Scaled Study

**DOI:** 10.1371/journal.pone.0084870

**Published:** 2013-12-26

**Authors:** Je Sung You, Hye-Jeong Lee, Yong Eun Chung, Hye Sun Lee, Myo Jeong Kim, Sung Phil Chung, Myeong-Jin Kim, Incheol Park, Ki Whang Kim

**Affiliations:** 1 Department of Emergency Medicine, Severance Hospital, Yonsei University College of Medicine, Seoul, Republic of Korea; 2 Department of Radiology, Severance Hospital, Research Institute of Radiological Science, Yonsei University College of Medicine, Seoul, Republic of Korea; 3 Department of Research Affairs, Biostatistics Collaboration Unit, Yonsei University College of Medicine, Seoul, Republic of Korea; Kagoshima University Graduate School of Medical and Dental Sciences, Japan

## Abstract

In contrast to patients with underlying cancer or chronic disease, injury patients are relatively young, and can be expected to live their natural lifespan if injuries are appropriately treated. Multiple and repeated diagnostic scans might be performed in these patients during admission. Nevertheless, radiation exposure in injury patients has been overlooked and underestimated because of the emergent nature of such situations. Therefore, we tried to assess the cumulative effective dose (cED) of injury patients in the emergency department. We included patients who visited the emergency department (ED) of a single tertiary hospital due to injury between February 2010 and February 2011. The cED for each patient was calculated and compared across age, sex and injury mechanism. A total of 11,676 visits (mean age: 28.0 years, M:F = 6,677:4,999) were identified. Although CT consisted of only 7.8% of total radiologic examinations (n=78,025), it accounted for 87.1% of the total cED. The mean cED per visit was 2.6 mSv. A significant difference in the cED among injury mechanisms was seen (*p*<0.001) and patients with traffic accidents and fall down injuries showed relatively high cED values. Hence, to reduce the cED of injury patients, an age-, sex- and injury mechanism-specific dose reduction strategy should be considered.

## Introduction

Recently, diagnostic X-ray imaging, including conventional radiography, computed tomography (CT) and nuclear medicine, have played major roles in screening, diagnosis and monitoring of treatment response in the evaluation of disease. Consequently, there has been a dramatic increase in radiation exposure: two-fold in per capita radiation dosage (from 3.6 mSv to 6.2 mSv) and six-fold in annual dosage from medical radiation (from 0.54 mSv to 3 mSv) in the United States (US) population between the early 1980s and 2006 [1,2]. Similar to rates observed in the general population, radiation exposure in injury patients who visited an emergency department (ED) has continuously increased; this rise is mainly due to the fact that CT has become one of the most important tools in the evaluation and planning for appropriate injury treatment [3,4,5]. 

Several studies on the cumulative effective dose (cED) of patients with a specific disease have been conducted [6,7,8]. Recently, a large-scale retrospective cohort study demonstrated an increased incidence of leukemia and brain tumors in patients who received low-dose radiation during childhood: cumulative dosages of 50 mGy and 60 mGy might triple the risk of leukemia and brain tumors, respectively [9]. However, populations in these studies included a high proportion of relatively older patients or patients who had underlying cancer or chronic disease, and as a result, the expected residual lifespan was relatively short. 

In comparison with cancer or chronic disease patients, injury patients are relatively young, and can live out their natural expected lifespan if their injuries are appropriately treated. Even though multiple and repeated scans might be performed during admission, radiation exposure in injury patients has been overlooked and underestimated because of it occurring during emergent situations. Therefore, the purpose of this study was to conduct a comprehensive analysis of radiation exposure in injury patients who visited the ED of a tertiary teaching hospital according to injury mechanism, type of radiological imaging, age, and sex. We expect this analysis to present an overview of radiation exposure in injury patients and to become a basis for finding possible ways to reduce radiation dose in injury patients.

## Methods

### Study design and clinical setting

This study was a retrospective study conducted in the ED of a tertiary teaching hospital with an annual load of approximately 65,000 patients. One conventional radiograph room and one CT room were constructed in the ED and are in operation seven days a week, 24 hours a day. This study was approved by the Institutional Review Board of our institution (Yonsei University College of Medicine), and the need for written informed consent from the participants was waived by the Institutional Review Board.

### Patients and injury mechanisms

Patients who visited our ED and registered for the National In-depth Injury Surveillance System between February 2010 and February 2011 were included in this study. Patients who were transferred out to other hospitals or who were transferred into our hospital were excluded because additional radiological examinations could have been performed at other hospitals. Through careful chart and image reviews, injury mechanisms were subdivided by modifying the injury mechanism of the Advanced Trauma Life Support® (ATLS®) for doctors (Table 1) [10]. If one patient visited with different injury mechanisms, their injuries were counted separately.

**Table 1:** Proportions of injury mechanisms.

**Injury mechanism**	**(n = 11676)**		
Blunt trauma (%)	8814 (75.5)	Vehicular impact when the patient is inside vehicle (%)	622 (5.3)
		Pedestrian injury (%)	468 (4.0)
		Injury to motorcyclist (%)	158 (1.4)
		Injury to cyclist (%)	313 (2.7)
		Assaults (intentional injury) (%)	1265 (10.8)
		Fall down injury (%)	699 (6.02)
		Blast injury (%)	1(0)
		Slip down injury (%)	4595(39.4)
		Injury by blunt object (%)	693 (5.9)
Penetrating injury (%)	12 (0.1)	Knife (%)	10 (0.1)
		Gun (%)	1 (0)
		Foreign object (%)	1 (0)
Simple laceration (%)	620 (5.3)		
Burn (%)	376 (3.2)		
Hanging (%)	9 (0.1)		
Drug Intoxication (%)	160 (1.4)		
Animal bite (%)	176 (1.5)		
Foreign body in airway track or alimentary track (%)	981 (8.4)		
Crushing injury by machines (%)	237 (2.0)		
Dislocation (jaw or shoulder dislocation, pulled elbow) (%)	291 (2.5)		

Note: Data in parentheses are the proportion of patient numbers

### Number of examinations

The total numbers of conventional radiographs and CT scans obtained during ED admission were recorded for each patient. For conventional radiographs that had different views, each view was counted separately (for example, flat and upright abdominal views were recorded as two studies, although they are usually performed simultaneously). In terms of CT scanning, one examination was recorded as one study despite the different phases it may have contained according to protocols. 

### Estimation of the effective dose

The effective dose was defined by the International Commission of Radiological Protection (ICRP) as an estimate of the corresponding uniform whole-body dose when non-uniform irradiation was developed, such as medical imaging [11,12]. The effective dose is commonly used as an assessment tool for radiation exposure because it can provide a relative value of radiation exposure between different types or ranges of radiological examinations regardless of target organ or body part [13]. In this study, the effective dose was used to compare radiation exposure in injury patients according to age, sex, injury mechanism, and different type of radiological examination. Compared to other studies [13,14,15,16], which usually use an average estimated dosage (for example, 10 mSv for all abdominal CT scans), one of the strong points in this study was that a patient-specific estimated dose was calculated for all CT scans. This process is a more accurate method compared to previous studies because the estimated dose during CT scanning can vary according to CT protocol and patient body mass index (BMI). 

Most recent CT scanners provide dose-length product (DLP) values for each examination, and the effective dose can be calculated with the following equation using these DLP values and body region-specific conversion factors (EDLP): 

Effective dose (mSv)=DLP*EDLP

We adapted the EDLP proposed by the National Radiological Protection Board (NRPB) report [17]. For patients younger than 20 years of age, the NRPB report only suggested EDLP values for 0, 1, 5, and 10-year-olds; therefore, EDLP values that were not presented in the NRPB report were calculated by log-linear interpolation using the suggested age values of 1, 2, 5, 10 and 20 years. 

Because radiation exposure is not measured study by study or patient by patient in conventional radiographs, the effective dose of conventional radiographs was adapted from the National Radiological Protection Board (NRPB) report [18]. If there was no effective dose suggested for a specific examination, the most similar examination dose was used (for example, the effective dose of town’s view was adapted from that of a facial bone radiograph).

### Statistical analysis

An analysis of variance (ANOVA) was used to compare the cED according to injury mechanism. An analysis of covariance (ANCOVA) with post-hoc analysis was used for age and sex adjusted multivariate analysis of cED according to injury mechanism. A *p*-value of less than 0.05 was considered to indicate statistical significance. Statistical analyses were performed using the Statistical Package for the Social Sciences (SPSS) version 18.0.0 (SPSS Inc., Chicago, IL, US).

## Results

### Patients

A database search identified 11,919 visits and 243 patients were excluded due to transfers out to other hospitals (n = 96) and transfers in from other hospitals (n = 147). Finally 11,676 patients were included in this study. Of these patients, 236 patients visited the emergency department twice, 14 visited three times, and 3 visited four times for different injuries. In terms of per-visit patient demographics, the average age was 28.0 years (range, 0-102 years; male:female, 6677:4999). The numbers of patients according to sex and age are summarized in Table 2. Patient numbers showed a bimodal distribution, and there were two peaks at 0-4 years (n = 2,487) and 25-29 years (n = 1,159). Detailed numbers and proportions of patients according to injury mechanism are presented in Table 1.

**Table 2:** The mean cumulative effective dose (mSv) according to patient age and sex.

			**The mean cED (mSv)**	
**Age**	**The number of patients (Male : Female)**	**All**	**Male**	**Female**
0-4	2487 (1509:978)	0.9 (0.0)	1.0 (0.0)	0.8 (0.1)
5-10	1044 (671:373)	0.9 (0.1)	1.0 (0.1)	0.9 (0.2)
11-14	513 (371:142)	1.1 (0.2)	1.3 (0.2)	0.8 (0.1)
15-19	612 (438:174)	3.8 (0.7)	4.2 (0.9)	3 (0.8)
20-24	991 (511:480)	1.7 (0.3)	2.2 (0.4)	1.1 (0.3)
25-29	1219 (655:564)	1.9 (0.3)	2.4 (0.5)	1.3 (0.2)
30-34	857 (479:378)	2.5 (0.4)	3.2 (0.6)	1.5 (0.4)
35-39	696 (389:307)	2.7 (0.4)	3.8 (0.7)	1.3 (0.3)
40-44	513 (324:189)	4.8 (0.8)	5.0 (1.0)	4.5 (1.2)
45-49	438 (263:175)	3 (0.5)	3.8 (0.8)	1.9 (0.5)
50-54	500 (260:240)	4.8 (0.8)	7.1 (1.4)	2.4 (0.6)
55-59	415 (208:207)	4.9 (1.1)	5.5 (1.6)	4.4 (1.5)
60-64	346 (157:189)	4.5 (0.6)	4.9 (1.0)	4.2 (0.8)
65-69	347 (157:190)	5.4 (0.7)	5.7 (1.0)	5.3 (0.9)
70-74	296 (123:173)	7.2 (1.0)	8.6 (2.0)	6.1 (0.9)
75-79	205 (89:116)	5.6 (0.7)	6.1 (1.1)	5.2 (0.9)
80-84	108 (44:64)	6.6 (1.1)	5.7 (1.8)	7.2 (1.5)
85-89	56 (17:39)	7.1 (1.4)	8.0 (2.8)	6.8 (1.6)
90-94	26 (11:15)	3.7 (0.8)	4.5 (1.4)	3.0 (1.0)
95-100	6 (1:5)	2.1 (0.8)	2.0	2.1 (1.0)
100-105	1 (0:1)	0.9	-	0.9

Note: Data in parentheses in the mean cED column are standard error; cED, the mean cumulative effective dose

### Number of radiologic examinations

**Figure 1:**
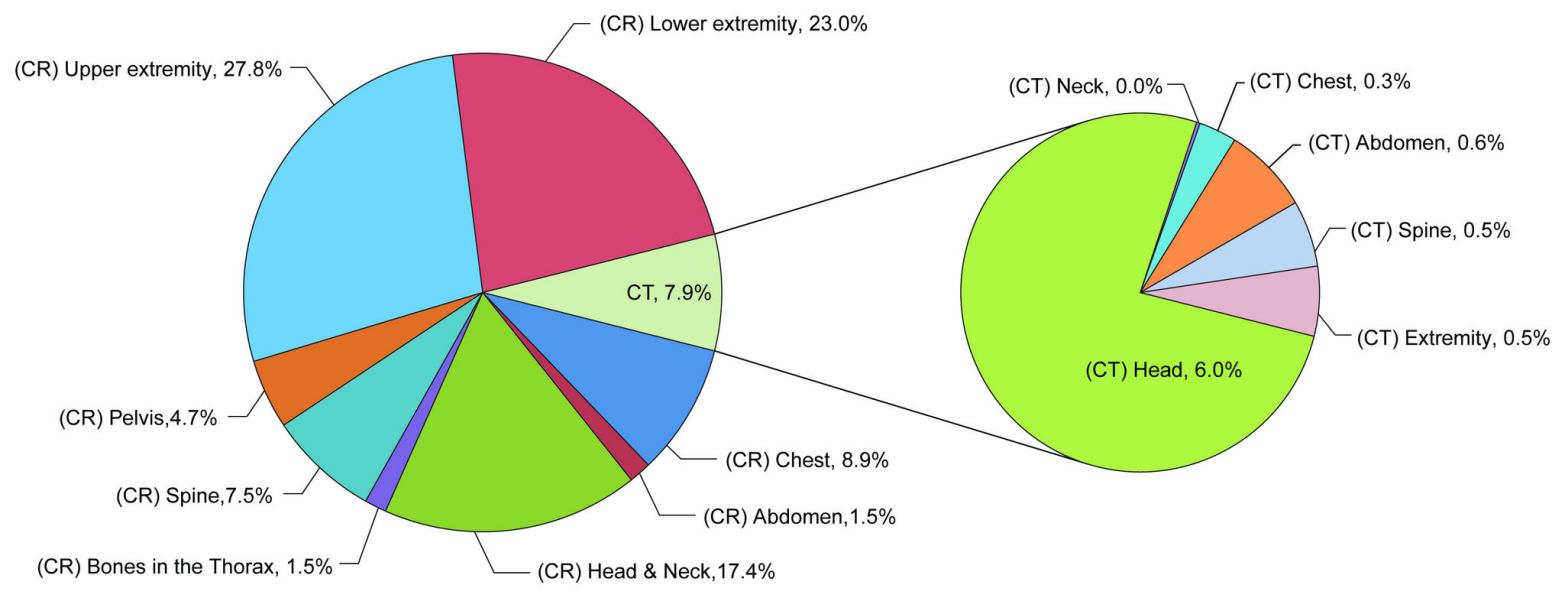
Proportion of radiological examinations (n = 78,025). CR: conventional radiography; CT: computed tomography.

Of the 11,676 visits, at least one conventional radiograph was performed in 7,868 visits (67.4%), and at least one CT scan was conducted in 4,175 visits (38.5%). No radiological examination was performed in 27.6% of patients. In all, 78,025 radiologic examinations were carried out. Among these, 92.2% (6.2 per visit) were conventional radiographs, and 7.8% (0.5 per visit) were CT scans (Figure **1**). 

### Cumulative effective dose

The cED of conventional radiographs consisted 12.7% of the total cED (3,891.0 mSv of 30,239.4 mSv), and that of CT was 87.1% (26,348.4 mSv of 30,239.4 mSv). The average cED per visit was 2.6 mSv for all patients and 6.3 mSv for those who had at least one radiological examination. When including the 28% of patients who did not undergo radiological examination, 67% of all the patients received less than 1 mSv per visit. Of all the studied patients, 2.7% received more than 20 mSv, and 27 of 11,676 (0.2%) received more than 100 mSv (Table **3**). 

**Table 3:** Distribution of the mean cumulative effective dose per visit.

**Mean cED** (**mSv**)	**Age: 0-4**	**5-9**	**10-14**	**15-19**	**20-59**	**60-74**	**75+**
0 (n=3218)	501:369	175:114	77:45	75:46	777:847	74:75	17:26
0>, 1<= (n=4583)	588:378	286:179	166:62	195:76	1242:983	126:201	38:63
1>, 3<= (n=2286)	217:126	158:54	94:27	101:31	617:510	112:132	41:66
3>, 20<= (n=1272)	202:105	51:25	33:8	49:14	325:147	89:101	53:70
20>, 100<= (n=290)	1:0	1:1	1:0	15:7	113:46	34:43	13:15
100> (n=27)	-	-	-	3:0	15:7	2:0	-

Patients were divided into subgroups based on age.

Note: Data in numbers are the number of patients (Male:Female)

A significant difference in the cED among injury mechanisms was seen (ANOVA*, p*<0.001 and ANCOVA, *p*<0.001). Among injury mechanisms that were the cause of ER visitations in more than 50 patients, the mean cED per visit was significantly higher in patients with injuries related to any kind of traffic accident (such as vehicular impact with the patient inside the vehicle, pedestrian injury, injury to motorcyclist, and injury to cyclist) and with fall down injuries than in other patients (ANCOVA with post-hoc analysis, *p*<0.001), whereas most patients with simple lacerations, burns or animal bites did not even receive any radiological examination (Table **4**). The detailed cED for each injury mechanism is presented in Table 5. Among patients who received more than 100 mSv, the most common cause was pedestrian injury (n=9), followed by falling injury (n=8), injury to a motorcyclist (n=5), vehicular impact with the patient inside the vehicle (n=2), slip down injury (n=2), and crushing injury involving machinery (n=1) (Table **4**).

**Table 4:** Distribution of the mean cumulative effective dose per visit (mSv) according to injury mechanism.

**Mechanism (%)**	0 mSv	>0 mSv, ≤1 mSv	>1mSv, ≤3mSv	>3mSv, ≤20mSv	>20mSv, ≤100mSv	>100
Vehicular impact when the patient is inside vehicle	67 (10.8)	173 (27.8)	226 (36.3)	98 (15.8)	56 (9.0)	2 (0.3)
Pedestrian injury	48 (10.3)	198 (42.3)	85 (18.2)	68 (14.5)	60 (12.8)	9 (1.9)
Assaults (intentional injury)	276 (21.8)	442 (34.9)	382 (30.2)	149 (11.8)	16 (1.3)	0
Fall down injury	78 (11.2)	204 (29.2)	181 (25.9)	181 (25.9)	47 (6.7)	8 (1.1)
Slip down injury	886 (19.3)	1915 (41.7)	1119 (24.4)	602(13.1)	71 (1.5)	2 (0.0)
Simple laceration	465 (75.0)	137 (22.1)	9 (1.5)	8 (1.3)	1 (0.2)	0
Burn	327 (87.0)	32 (8.5)	14 (3.7)	3 (0.8)	0	0
Animal bite	141 (80.1)	33 (18.8)	2 (1.1)	0	0	0

Note: Data are the number of patients and data in parentheses are the proportion of patient numbers for each specific injury mechanism..

**Table 5:** The mean cumulative effective dose (mSv) according to injury mechanism.

**Mechanism (%)**	**Age: 0-4 (2487)**	**5-9 (1044)**	**10-14 (513)**	**15-19 (612)**	**20-59 (5629)**	**60-74 (989)**	**75+ (402)**
Vehicular impact whenthe patient is inside vehicle (%)	1.3 (24)	5.7 (19)	1.6 (8)	9.4 (25)	5.6 (464)	10.9 (74)	14.3 (8)
Pedestrian injury (%)	3 (20)	2.3 (50)	1.9 (17)	14.5 (36)	12.5 (255)	14.6 (57)	10.8 (33)
Injury to motorcyclist (%)	1.6 (4)	0.1 (1)	0.8 (3)	16.2 (32)	14 (111)	2.5 (6)	46.2 (1)
Injury to cyclist (%)	1.3 (22)	1.3 (58)	5.1 (26)	1.5 (24)	2.6 (149)	7.4 (29)	5.2 (5)
Assaults (intentional injury) (%)	0.5 (67)	0.8 (86)	1.4 (102)	2.1 (148)	1.7 (798)	5.6 (52)	2.6 (12)
Fall down injury (%)	2.2 (337)	1.6 (85)	1.8 (28)	25.2 (17)	16.2 (166)	14.9 (46)	11.2 (20)
Slip down injury (%)	1.1 (1156)	0.8 (476)	0.9 (207)	1.3 (189)	1.9 (1770)	4.8 (523)	5.3 (274)
Injury by blunt object (%)	0.7 (145)	0.8 (66)	0.6 (28)	0.9 (33)	0.7 (378)	1.4 (37)	3.3 (6)
Simple laceration (%)	0.1 (82)	0.1 (22)	0 (20)	0.7 (26)	0.1 (444)	0.1 (22)	0 (4)
Burn (%)	0 (118)	0 (21)	0 (7)	0 (10)	0.1 (203)	0.6 (12)	1.1 (5)
Drug intoxication (%)	0.1 (4)	0 (1)	None	0.1 (12)	1.4 (116)	1.5 (21)	0.1 (6)
Animal bite (dog, cat) (%)	0 (17)	0.1 (12)	0.3 (8)	0 (6)	0 (110)	0 (19)	0.2 (4)
Foreign body (%)	0.1 (168)	0.1 (94)	0.1 (45)	0.1 (48)	0.1 (537)	1.4 (72)	3.2 (17)
Crushing injury (%)	0 (93)	0 (31)	0 (8)	0 (4)	2.4 (85)	0 (12)	0.4 (4)
Jaw dislocation, pulled elbow (%)	0 (230)	0.2 (22)	0.4 (5)	0 (1)	0.1 (28)	0.2 (4)	0 (1)

Patients were divided into subgroups based on age.

Note: Data are the mean cumulative effective dose in each patient. Data in parentheses are the number of patients. Injury mechanisms with 50 patients or less in total number were not provided in this table.

### Subgroup analysis of patients by age

The mean cED per visit was relatively higher in patients between 15-19 years old (juveniles), between 40-44 years old (middle-age adults), and between 70-74 years old (senescence), compared to other age groups (Table **2**). In patients in the 0-14 years age group, the most common injury mechanism was slip down injury. The cED of these injury mechanisms was between 30.8% and 54.2% of the total cED (Table **6**). Of all the CT scans performed, head CT contributed to 65.8%-86.1% of the total cED (Table **7**). In patients in the 15-56 years age range, the proportion of injury mechanism was more broadly and similarly distributed among injury mechanisms. In patients who were more than 60 years old, the proportion of slip down injury increased again. The proportions of certain types of radiological examinations and exam type-specific cED were similar in patients who were older than 15 years old. (Table **7**).

**Table 6:** The total cumulative effective dose (mSv) according to the injury mechanism in age-specific subgroups.

**Mechanism (%)**	**Age: 0-4 (2487)**	**5-9 (1044)**	**10-14 (513)**	**15-19 (612)**	**20-59 (5629)**	**60-74 (989)**	**75+ (402)**
Vehicular impact whenthe patient is inside vehicle (%)	31.9 (1.4)	107.7 (11.1)	12.5 (2.1)	234.3 (10.0)	2617.8 (16.2)	807.1 (14.5)	114.7 (4.8)
Pedestrian injury (%)	59.4 (2.6)	116.0 (11.9)	32.6 (5.6)	522.5 (22.2)	3185.8 (19.8)	833.5 (15.0)	356.8 (15.0)
Injury to motorcyclist (%)	6.3 (0.3)	0.1 (0.0)	2.5 (0.4)	519.4 (22.1)	1551.9 (9.6)	15.1 (0.3)	46.2 (1.9)
Injury to cyclist (%)	28.5 (1.3)	77.8 (8.0)	133.8 (23.0)	35.7 (1.5)	380.7 (2.4)	214.2 (3.8)	25.9 (1.1)
Assaults (intentional injury) (%)	31.6 (1.4)	71.7 (7.4)	138.8 (23.8)	306.1 (13.0)	1371.9 (8.5)	293.1 (5.3)	31.5 (1.3)
Fall down injury (%)	724.8 (32.3)	138.9 (14.3)	51.7 (8.9)	428.1 (18.2)	2696.8 (16.7)	684.7 (12.3)	224.4 (9.5)
Slip down injury (%)	1215.3 (54.2)	395.3 (40.6)	179.7 (30.8)	249.2 (10.6)	3324.0 (20.6)	2511.8 (45.1)	1439.3 (60.7)
Others (%)	146.4 (6.5)	66.7 (6.8)	30.9 (5.3)	57.7 (2.5)	1019.6 (6.3)	206.2 (3.7)	132.7 (5.6)

Note: Data are the total cumulative effective dose. Data in parentheses are the propotion of cED according to total cED in specific age group. Injury mechanisms other than the top seven mechanisms with the highest total cEDs were grouped together as ‘Others’ and sum values were provided.

**Table 7:** Types and number of CT examinations and their contributions to the total cED.

**Types of CT**	**Age: 0-4**	**5-9**	**10-14**	**15-19**	**20-59**	**60-74**	**75+**
Head	761 (86.1)	353 (70.4)	210 (65.8)	312 (22.5)	2253 (21.1)	584 (18.3)	209 (16.3)
Neck	0 (0)	0 (0)	0 (0)	3 (0.6)	13 (0.4)	1 (0.1)	1 (0.1)
Chest	2 (0.3)	2 (0.7)	3 (4.6)	9 (3.2)	108 (5.5)	54 (6.8)	27 (6.3)
Abdomen	2 (0.8)	7 (7.0)	2 (8.1)	46 (53.7)	246 (48.9)	111 (50.0)	65 (45.5)
Spine	1 (0.4)	10 (4.7)	5 (5.5)	20 (6.39)	223 (11.5)	77 (11.0)	29 (10.3)
Extremity	1 (0)	4 (0.1)	11 (1.5)	25 (1.5)	221 (0.8)	72 (0.8)	29 (2.3)

Note: Data in parentheses are the proportion of cED of a specific type CT in total cED (%)

## Discussion

Our results showed that, although the number of CT scans was relatively small compared to conventional radiography, most of the cED of injury patients resulted from CT. Also, traffic accident-related injury patients and fall down injury patients received relatively high radiation exposure, whereas many patients with simple lacerations, burns or animal bites did not undergo any radiological examination during their ED visit. In patients who were less than 15 years old, the most common injury mechanism was slip down injury, and most of the cED resulted from head CT scanning. 

As expected, the most common injury mechanism in patients in the 0-4 years age group was slip down injury, which accounted for 46.5% (1156 of 2487 patients) of all injury mechanisms and 54.2% of the total cumulative effective dose, followed by fall down injury (13.6% of injury mechanisms, 32.3% of the total cED). This finding might be because toddlers do not walk in a stable manner, and they are frequently injured by slip downs or fall downs. Infants often fall from high locations, such as beds; therefore, fall down injuries might make up a large proportion of injury mechanisms. Furthermore, in infants and toddlers, the head is relatively larger in proportion to both weight and body length than it is in older patients; a reason why injury to the head is thought to be more common in this age group than in older patients. This trend might explain why almost all CT scans (761 of 767, 99.2%) performed in patients between 0-4 years old were head CT scans. 

A previous study reported that there were differences in cED depending on trauma mechanism[15]. In that study, patients with traffic accident related injuries (motor vehicle collision, bicycle, pedestrian collision) and with fall down injuries showed higher cED than that of other patients, which was comparable with our result. In comparision to our study, patients with burn also showed high cED in the previous study. This might be because the demographics of included patients were different with only pediatric trauma patients being included in the previous study. The reason why patients with traffic accident related injuries or with fall down injuries have higher cED than other patients is probably because patients with these kind of injury mechanisms are more often severely injured than others [19,20], and diagnostic radiologic modalities might have to be more frequently performed for these severely injured patients. 

Nevertheless, the first and also the most effective way to reduce radiation exposure is to avoid performing unnecessary examinations. According to an informal poll of pediatric radiologists who were surveyed during a multidisciplinary conference, organized by the Society for Pediatric Radiology, about 30% of CT examinations might not be necessary [21]. An awareness of the possible radiation hazards present during radiological examinations is essential for the risk-benefit evaluation of radiological examinations, and an education campaign for physicians and patients might be one solution to increase understanding of the possible hazards of low-level radiation [22,23]. In addition, appropriate guiding criteria that recommends when CT examinations can be justified could also reduce unnecessary scanning. In this aspect, the American College of Radiology appropriateness criteria has been shown to potentially reduce the estimated radiation dose by 44% when correctly applied [24]. Second, if a CT is performed for patient evaluation, the scanning protocol must be adjusted to the individual patient. With the advent of multidetector-row CT (MDCT), multiphase CT is frequently performed. Obtaining additional phase CT images directly increases radiation exposure, so only diagnostically beneficial phase scans should be obtained. In addition, scanning area (field of view) optimization could reduce unnecessary radiation exposure. The field of view (FOV) should be limited only to the organs or areas of interest. If a chest or abdomen evaluation is needed, omitting the thyroid or gonads from the FOV can reduce unnecessary radiation exposure on these radiosensitive organs. Third, if technically feasible, state-of-the-art dose reduction techniques, such as automatic tube current/voltage modulation, and image noise reduction with iterative reconstruction, should be applied. If these techniques can be used together, more than half of the radiation exposure can be reduced without sacrificing diagnostic accuracy [25]. Fourth, the development and use of alternative imaging modalities for injury patients (i.e., magnetic resonance image (MRI) or contrast-enhanced ultrasonography) can reduce radiation exposure. According to a recently published report, access to alternative diagnostic modalities allows for a decreased number of CTs among pediatric patients who visit the ED [26]. Fifth, clinical assessment criteria rather than a radiologic examination could present an alternative treatment or diagnosis plan. As presented in our results, performing a head CT is a major source of radiation exposure in children. According to a previous report, 70-90% of treated brain injuries are mild [27], and not all of them require CT examinations. Recently, age-specific prediction rules for identifying children at very low risk of clinically important traumatic brain injuries (and for whom CT is unnecessary) were validated with a high negative predictive value (99.95%-100%) and sensitivity (96.8%-100%) [28]. If this kind of clinical assessment model can be widely distributed, the number of CT scans and therefore the amount of radiation exposure can be reduced. 

There are several limitations in our study. First, this study had a retrospective design with a single center experience. Second, other imaging studies, such as fluoroscopy and angiography, were not included in this study and the cED might be underestimated, especially in patients with severe trauma. Third, we did not evaluate the effect of CT scanning parameters in each patient, which can affect the radiation exposure during the CT scan. CT protocol optimization is also important and an effective way to reduce radiation exposure. Hence, further studies on optimizing CT protocols in injury patients should be done in the future. The last limitation is the exclusion of patients because transfers in or out of the hospital might affect the results of this study, although the number of such patients was relatively small compared to the total number of included patients.

In conclusion, most of the cED of injury patients resulted from CT, in spite of the relatively small proportion of examination numbers and the mean cED was different according to injury mechanism, age and sex. Therefore, to reduce the cED of injury patients, age-, sex- and injury mechanism-specific dose reduction strategies should be considered in more detail in the future.
